# Incorporation of reporter genes into mitochondrial DNA in budding yeast

**DOI:** 10.1016/j.xpro.2022.101359

**Published:** 2022-05-25

**Authors:** Magdalena Rzepka, Tamara Suhm, Martin Ott

**Affiliations:** 1 Department of Biochemistry and Biophysics, Stockholm University, 10691 Stockholm, Sweden; 2 Department of Medical Biochemistry and Cell Biology, University of Gothenburg, 41390 Gothenburg, Sweden

**Keywords:** Cell Biology, Genetics, Model Organisms, Molecular Biology

## Abstract

Many aspects of mitochondrial gene expression are still unknown, which can be attributed to limitations in molecular tools. Here, we present a protocol to introduce reporter genes into the mitochondrial genome of budding yeast, *Saccharomyces cerevisiae*. Mitochondrially encoded reporter constructs can be used to interrogate various aspects of mitochondrial gene expression. The power of this technique is exemplified by a mitochondrially encoded nanoluciferase, which allows to monitor levels of mitochondrial translation under a variety of growth conditions.

## Highlights

• Engineering reporter genes for mitochondrial gene expression
• A genetic reporter is inserted as an additional gene in mitochondrial DNA
• Transfer of mitochondrial DNA molecules between yeast strains
• A versatile approach to study mitochondrial protein biogenesis in living cells

## Abstract (editor highlights)

Publisher’s note: Undertaking any experimental protocol requires adherence to local institutional guidelines for laboratory safety and ethics.

## Graphical abstract

**Figure:**
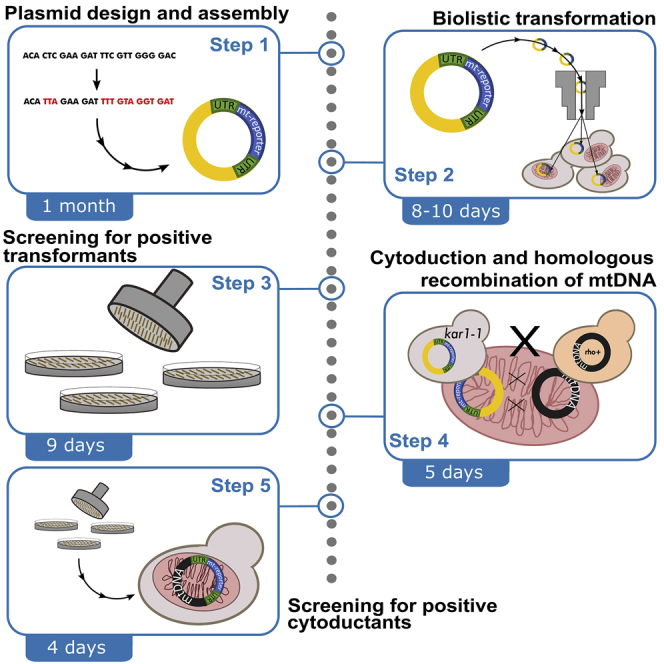

## Before you begin

**Figure 1:**
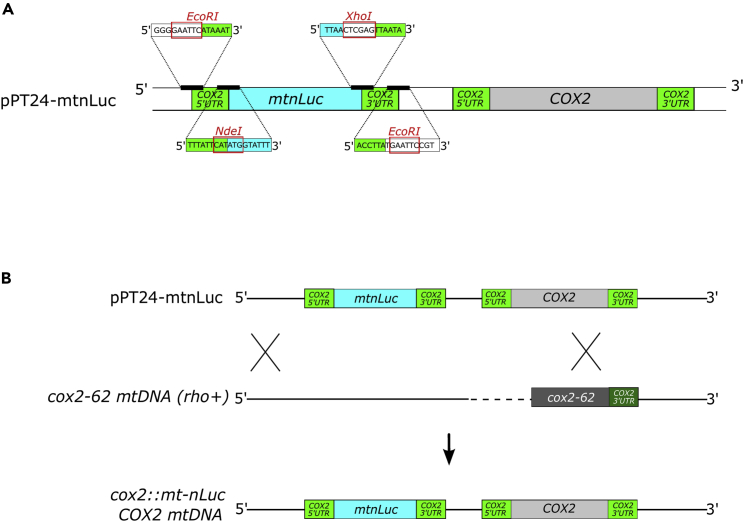
Integration of *mtnluc* into mitochondria genome (A) Schematic of the plasmid pPT24-mtnLuc that contains *mtnuc* sequence flanked by *COX2* UTRs cloned into the pPT24 plasmid using *EcoRI* restriction digestion site. (B) Schematic representation of *mtnluc* integration into mitochondrial genome via homologous recombination.

Here we present an approach to incorporate reporter genes into mitochondrial DNA (mtDNA) of budding yeast. Originally, a method was developed in the laboratory of Tom Fox to integrate a reporter gene into a certain open reading frame of the mitochondrial genome (Steele et al., 1996). For this, yeast cells were transformed with a biolistic gene delivery system, followed by screening for a reporter activity. The exchange of an authentic open reading frame to that of the reporter caused inability to perform oxidative phosphorylation with consequences for cellular physiology. Later, the Tom Fox group developed a plasmid, pPT24, which allowed to express a recoded open reading frame in mitochondria as additional gene (Mireau et al., 2003; Thorsness and Fox, 1993). The principle of the latter approach is to incorporate the reporter gene in an otherwise silent region of the mtDNA (Figure 1A). For this, plasmid pPT24 has a unique *EcoR*I site that allows to clone in a fully functional gene, encompassing a standard promoter driving the expression of an mRNA containing the 5′-untranslated regions (UTRs) of *COX2* (Gruschke et al., 2012). Moreover, the plasmid contains a large portion of the upstream region of *COX2* including its open reading frame.

**Figure 2:**
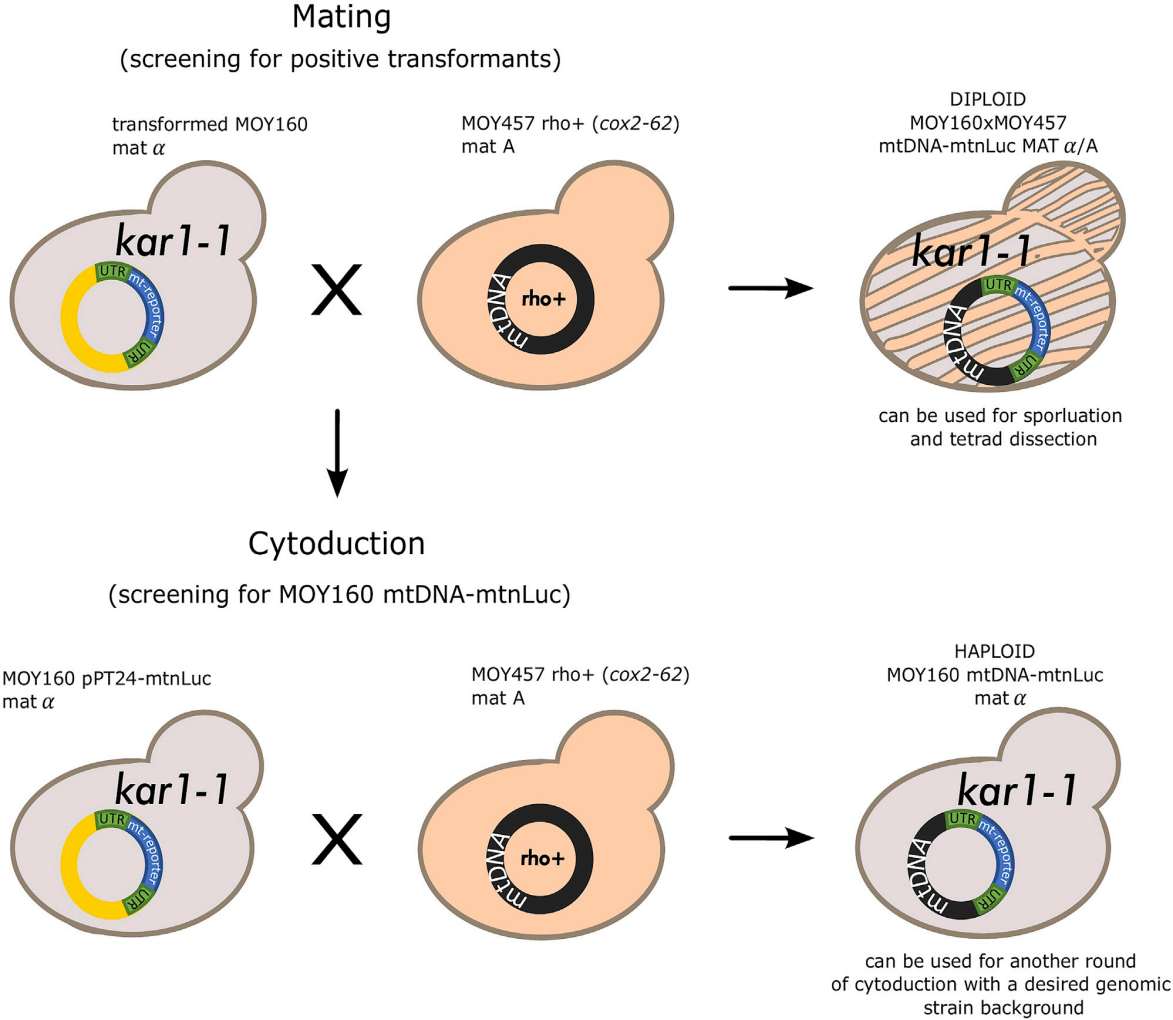
Schematic representation of the genetic manipulations to obtain a yeast strain with mtDNA coding for all 8 authentic proteins and the additional reporter protein (mtDNA-mtnLuc) The first step requires mating of the colony that underwent biolistic transformation and a *rho*^*+*^ strain to screen for colonies that contain the pPT24-mtnLuc plasmid in mitochondria. In the second step, the colony with pPT24-mtnLuc is used for cytoduction with the *rho*^*+*^ strain to allow for homologous recombination of their mitochondrial genomes and obtain a haploid strain with mtDNA-mtnLuc.

In order to be functionally expressed, the reporter gene needs to be recoded to fit the mitochondrial codon usage and cloned into pPT24. The final plasmid is then delivered into the mitochondria via biolistic transformation where it can finally be recombined with a mutant mtDNA that carries a deletion in the *COX2* gene (Figure 1B). Identification of the transformants is a key aspect of the method. Here, a two-step procedure is used (Figure 2). First, general transformants are identified with the help of a nuclear plasmid that carries a selection marker cassette that reverts leucine auxotrophy. The plasmid is co-delivered with pPT24 into cells during the biolistic transformation. Next, mitochondrial transformants are identified by regain of respiratory growth. Because recombination restores the damaged *COX2* gene, cells that show respiratory competence have obtained the transformed plasmid (Suhm et al., 2018). In this protocol, we describe as an example the introduction of a gene encoding for nanoluciferase into mtDNA of *Saccharomyces cerevisiae*. In principle, this procedure can be used to integrate various reporter genes into mitochondrial DNA, allowing to analyze different molecular pathways.

### Reporter gene sequence and plasmid design

**Timing: 4 weeks**

This section deals with the preparation of the plasmid for homologous recombination in mitochondria. This plasmid (pPT24) carries the sequence upstream of the *COX2* locus, followed by the *COX2* original sequence. A unique *EcoR*I site was engineered into the silent region upstream of the *COX2* locus, into which the reporter gene can be integrated. Mitochondrial translation in yeast requires translational activators – proteins that interact with the UTRs of mRNAs (Costanzo and Fox, 1990) and that mediate their translation. In order to avoid changes in expression due to activated feedback loops that regulate expression of certain mRNAs (Ott et al., 2016), we have used *COX2* 5′-UTR driving the expression of the reporter. Hence, the sequence encoding the recoded nanoluciferase (mtnLuc) together with *COX2* UTRs was inserted into the *EcoR*I site of plasmid pPT24 that will allow the reporter to follow *COX2* expression in mitochondria.

To express the reporter gene from mitochondrial DNA, its nucleotide sequence has to be recoded to match mitochondrial codon usage. For this, a reverse translation of a protein sequence of interest can be obtained from a variety of online resources (for example https://www.genecorner.ugent.be/rev_trans.html), using codon usage from *S. cerevisiae* mtDNA). The recoded sequences can be synthesized commercially and are then integrated by standard cloning procedures into pPT24. Following those procedures, we designed a plasmid (pPT24-mtnLuc) containing a gene encoding for nanoluciferase that can be synthesized by mitochondrial ribosomes.

***Note:*** The synthesis of the recoded gene can take several weeks depending on the length of the nucleotide sequence.

***Note:*** Other 5′- and 3′-UTRs could be used as well to control expression of the reporter activity. However, the flanking sequences of *COX2* could be advantageous because they are rather short, which can decrease the probability of spontaneous rearrangements by recombination.

***Note:*** It can be advisable to check the sequence of the planned mRNA for the propensity to form secondary structures. If excessive structures are formed, it is possible that this decreases translation efficiency of the reporter. Therefore, it might be a good idea to exchange codons in this area with alternative, synonymous codons to reduce secondary structure formation.

### Plasmid isolation

**Timing: 2 days**

For the biolistic transformation, high yield and purity of plasmids pPT24-mtnLuc and pRS315 (used to identify nuclear transformants) are required. We used QIAGEN Plasmid Midi Kit according to the manufacturer’s protocol, but other high yield kits can likely be used as well. In the last step, the plasmids were eluted with 20 μL of sterile deionised H_2_O to yield a high concentration (around 7–10 μg/μL).

### Tungsten particles preparation

**Timing: 45 min**

The DNA will be delivered on tungsten particles that are accelerated to penetrate the yeast cells. Preparation of these tungsten-DNA particles is one key aspect for efficient transformation.

1. Mix 50 mg of tungsten powder with 1.5 mL of 70% ethanol.
2. Vortex and incubate at 22°C for 10 min.
3. Spin 15 s at 13,000 × *g*, discard supernatant and resuspend the particles in 1.5 mL of sterile deionised H_2_O.
4. Spin 15 s at 13,000 × *g*, discard supernatant and resuspend the particles in sterile 50% glycerol (16.7 μL/mg particles). The particles in glycerol can be store at −20°C for at least 4 years.

## Key resources table

**Table:** 

REAGENT or RESOURCE	SOURCE	IDENTIFIER
**Antibodies**		

Anti-nanoluciferase	This study	Home made
Anti-Aco1	This study	Home made
Anti-Cox2	This study	Home made

**Chemicals, peptides, and recombinant proteins**		

Tryptone/peptone	Carl Roth	8952.5
Yeast extract	Serva	24540.03
Dextrose	Sigma-Aldrich	G8270
Glycerol	VWR	24387.292
Raffinose	Sigma-Aldrich	R0250
Yeast nitrogen base	Invitrogen	Q30009
Ammonium sulfate	Merck	1012171000
Sorbitol	Carl Roth	6213.3
Agar	VWR	J637
L-Adenine	Sigma-Aldrich	A9126-25g
L-Arginine	AppliChem	A3675,0100
L-Uracil	AppliChem	A0667,0025
L-Leucine	AppliChem	A3460,0100
L-Lysine	AppliChem	A1342,0100
L-Histidine	AppliChem	A3733,0100
L-Tryptophan	AppliChem	A3445,0100
Tungsten powder, < 1 micron	AlfaAesar	44210
Calcium chloride	Sigma-Aldrich	223506
Spermidine	Sigma-Aldrich	S0226
Rupture disks	Bio-Rad	1652329
Macrocarriers	Bio-Rad	1652335
QIAGEN Plasmid Midi Kit	QIAGEN	12145

**Critical commercial assays**		

Nano-Glo® Luciferase Assay System	Promega	N1110

**Deposited data**		

pPT24	(Thorsness and Fox, 1993), available at Addgene	184419
pPT24-mtnLuc	This study, available at Addgene	184339

**Experimental models: Organisms/strains**		

MOY160: *S. cerevisiae* strain *Mat α, ade2-101, Δleu2, ura3-52, Δarg8::URA3, kar1-1; mtDNA: rho*^*0*^	(Steele et al., 1996)	N/A
MOY457: *S. cerevisiae* strain Mat a, *lys2, Δarg8::hisG, ura3-52, leu2-3,112; his3ΔHinDIII; mtDNA: cox2-62, rho*^*+*^	(Dunstan et al., 1997)	N/A

**Recombinant DNA**		

pPT24-mtnLuc	This study, available at Addgene	184339
pRS315	(Sikorski and Hieter, 1989)	ATCC 77144

**Other**		

Biolistic PDS-1000/He Particle Delivery System	Bio-Rad	165-2257
Luminometer	Promega	GM2000

## Materials and equipment

### Biolistic PDS-1000/He particle delivery system

**Figure 3:**
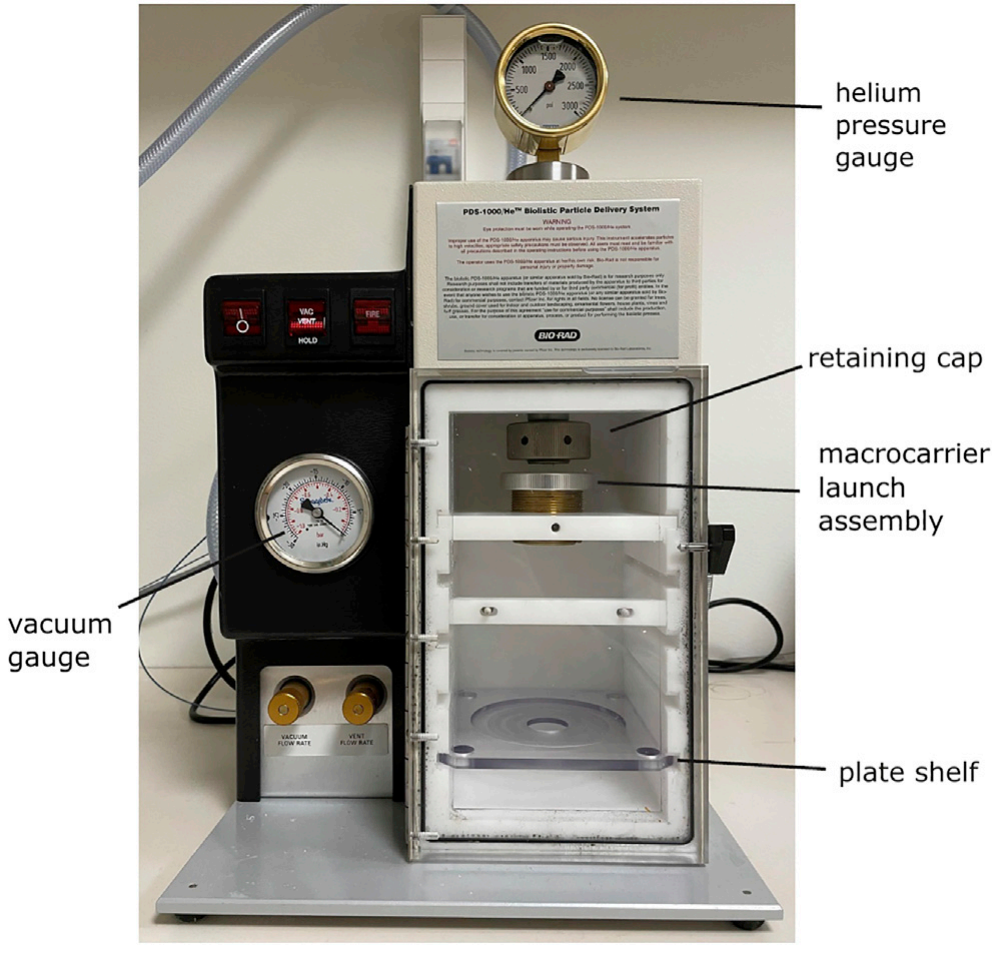
Explanatory picture presenting setup of biolistic transformation chamber with all the crucial components

This particle delivery system uses pressurized helium to introduce DNA-coated tungsten particles into cells at high velocity. The pressure can be built up thanks to a rupture disk that bursts at indicated pressure. Upon the burst, tungsten particles, previously spread on macrocarriers, are accelerated to penetrate the cells (Figure 3).

### Stock solution preparation

**Table:** YP media

Reagent	Final concentration (g/L)	Amount
Tryptone/peptone	20 g/L	20 g
Yeast extract	10 g/L	10 g
Deionized Water	n/a	Up to 1 L

***Note:*** Autoclave the same day after preparation. YP can be stored at 22°C for long term. Raffinose, dextrose or glycerol can be autoclaved separately before mixing with YP. Recommended stocks are 20%, 40% and 30%, respectively.

**Table:** Biolistic transformation plates, 500 mL (approximately 7 plates)

Reagent	Final concentration	Amount
Agar	30 g/L	15 g
Yeast nitrogen base	6.7 g/L	3.35 g
Sorbitol (2.4 M)	1 M	208 mL
Dextrose (40%)	5%	62.5 mL
Adenine	0.1 mg/mL	50 mg
Arginine	0.02 mg/mL	10 mg
Uracil	0.02 mg/mL	10 mg
Histidine	0.015 mg/mL	7.5 mg
Tryptophan	0.015 mg/mL	7.5 mg
Lysine	0.03 mg/mL	15 mg
Water	n/a	229.5 mL

***Note:*** Mix agar and amino acids with 218 mL of H_2_O and yeast nitrogen base in 11.5 mL of H_2_O. Put a magnetic stirrer into both bottles to facilitate mixing. Autoclave them separately and then mix with the rest of components. Prepare the plates at least three days in advance, so they can dry properly before the transformation.

***Note:*** Petri dishes for biolistic transformations need to be 100 mm × 15 mm to fit into the biolistic chamber. To facilitate screening of transformants we recommend using only plates of this size.

**Table:** Auxotrophic selection plates without leucine (SD-Leu), 500 mL (approximately 10 plates)

Reagent	Final concentration	Amount
Agar	20 g/L	10 g
Yeast nitrogen base	1.7 g/L	0.85 g
Ammonium sulfate	5 g/L	2.5 g
Dextrose (40%)	2%	25 mL
Adenine	0.02 mg/mL	10 mg
Arginine	0.02 mg/mL	10 mg
Uracil	0.02 mg/mL	10 mg
Histidine	0.015 mg/mL	7.5 mg
Tryptophan	0.015 mg/mL	7.5 mg
Lysine	0.03 mg/mL	15 mg
Water	n/a	475 mL

***Note:*** Mix agar, yeast nitrogen base, ammonium sulfate and amino acids with water. Put a magnetic stirrer into both bottles to facilitate mixing. Adjust pH to 5.5 with KOH and autoclave. Mix with autoclaved dextrose. Prepare at least one day in advance.

***Note:*** Follow this recipe omitting or adding different amino acids to prepare other auxotrophic selection plates.

**Table:** YP plates with dextrose or glycerol (YPD or YPG), 500 mL (approximately 10 plates)

Reagent	Final concentration (g/L)	Amount
Agar	2%	10 g
YP media	n/a	475 mL (for YPD) or 466 mL (for YPG)
Dextrose (40%) or glycerol (30%)	2%	25 mL (for YPD) or 34 mL (for YPG)

***Note:*** Dextrose and glycerol are autoclaved separately. Mix agar with 200 mL of YP medium and autoclave. Then add the rest of the YP medium and carbon source.

## Step-by-step method details

### Biolistic transformation of the yeast cells

**Timing: 8–10 days**

In this step, a yeast strain lacking mitochondrial DNA (MOY160) is transformed with two plasmids pRS315 and pPT24-mtnLuc for nuclear and mitochondrial transformation. The plasmids transformed into mitochondria are replicated and maintained (Fox et al., 1988). Detailed instructions on how to use the biolistic particle delivery system can be found in the manufacturer’s manual (https://www.bio-rad.com/webroot/web/pdf/lsr/literature/M1652249.pdf).

1. Prepare yeast cells for biolistic transformation.
  a. Grow the yeast cells (MOY160) for around 15 h in YP+2% raffinose (YPR) at 30°C, 170 rpm.
  b. The following day dilute the culture to 0.05 OD_600_ in 30 mL of YPR and grow for 2 days at 30°C, 170 rpm. Cells should be in stationary phase. OD_600_ is expected to be around 3–4, depending on the incubation time.
  c. Harvest the cells by centrifugation at 4,000 × *g* for 5 min.
  d. Resuspend the pellet in 0.6 mL YP+2% dextrose (YPD) and plate very evenly 100 μL on seven biolistic transformation plates using sterile glass beads.
  e. Let the plates dry for 2–4 h at 30°C.

**CRITICAL:** If the plates are too wet, the vacuum in step 3 cannot build up.

2. Precipitation of DNA on tungsten particles (on ice).
  a. To a 1.5 mL tube add:
    i. 10–15 μg of plasmid pPT24-mtnLuc and 5 μg of the marker plasmid pRS315 (less than 15 μL in total),
    ii. 100 μL tungsten particles,
    iii. 40 μL 0.1 M spermidine.
    iv. 100 μL 2.5 M CaCl_2_.
  b. Incubate on ice for 10 min, vortex every 2 min.
  c. Spin 30 s at 13,000 × *g*, discard supernatant and resuspend particles in 200 μL 100% ethanol (ice-cold).
  d. Spin as previously, discard supernatant and resuspend particles in 200 μL 100% ethanol (ice-cold).
  e. Spin as previously, discard supernatant and resuspend particles in 60 μL 100% ethanol (ice-cold).
    ***Note:*** The tungsten particles need to be thoroughly resuspended before distributing them on the macrocarriers. We recommend vortexing the tube or triturating particles against the wall of the tube with a pipette tip.
    **CRITICAL:** It is important to work as sterile as possible. All the metal equipment (macrocarrier holders, tweezers) should be autoclaved/decontaminated.
  f. Insert six macrocarriers into macrocarrier holders with tweezers.
  g. Equally distribute the DNA-coated tungsten particles on the macrocarriers.
  h. Let particles dry for a short time (around 10–15 min).
3. Preparation of the biolistic chamber and bombardment (Figure 3).
  ***Note:*** The first bombardment should be performed without the target cells or tungsten particles.
  a. The biolistic chamber needs to be cleaned with ethanol and dried before use.
  b. Place the rupture disk into the recess of the retaining cap and mount it onto the end of the gas acceleration tube inside the biolistic chamber.
  c. Place the macrocarrier with the particles facing downwards into the macrocarrier launch assembly and secure with the cap.
  d. Insert the macrocarrier launch assembly into the chamber and adjust the distance by screwing in or out the assembly so the distance between the macrocarrier and the retaining cap is around 0.5 cm.
  e. Place the plate with yeast cells in the chamber on the target plate shelf and close the doors.
    ***Note:*** The distance between the macrocarrier launch assembly and the target plate shelf can be varied according to the results. In our experience shelves 2 or 3 from the top give the best results.
  f. Start vacuum by pressing VAC and wait until it reaches 28.5–29 inches of mercury.
  g. Switch the button from VAC to HOLD.
  h. Press FIRE and keep until the rupture disk bursts (around 1,100 psi) and helium pressure gauge drops to 0.
  i. Switch the button from HOLD to VENT.
  j. Recover the plate and remove the leftovers of the macrocarrier with sterile tweezers.
  k. Repeat the steps b–j with the remaining plates and macrocarriers.
  l. Incubate the cells for 5–7 days at 30°C.
    **Pause point:** The plates can be stored at 4°C for up to two weeks (optional).
    **CRITICAL:** The abovementioned levels of vacuum and pressure at which the rupture disk bursts are critical for successful transformation and might require testing for each setup.

### Screening for positive transformants

**Timing: 9 days**

The screening requires two steps. First, MOY160 cells that underwent the transformation with pRS315 plasmid are scored for growth on media lacking leucine. Secondly, the positive colonies are used for mating with another strain (MOY457) that has a disruption in the *COX2* gene making this strain respiratory deficient. The original sequence can be reintroduced from the pPT24-mtnLuc plasmid present in mitochondria of the transformed MOY160 strain colonies (here MOY160 pPT24-mtnLuc). This plasmid carries the full wild type *COX2* sequence. After mating and homologous recombination in mitochondria, the mtDNA regains the functional *COX2* sequence and the resulting strain is able to respire as scored by growth on plates containing glycerol (YP+ 2% glycerol, YPG).

4. Restreak at least 1,000 colonies from the biolistic plates to SD-Leu plates (approximately 100 colonies per plate). Incubate for 3 days at 30°C. Troubleshooting 1 and 2.
5. Transfer the colonies by replica plating onto masterplates (YPD plates) and incubate for 1 day at 30°C.

***Note:*** Masterplates are prepared as a back-up of the colonies picked up on the SD-Leu plates.

6. The same day start a culture of MOY457 in 50 mL of YPD and grow for around 15 h at 30°C, 170 rpm.
7. The next day expected OD_600_ of the cells is around 5–6. Harvest all the cells (4,000 × *g*, 5 min) and plate them using glass beads on the appropriate number of YPD plates. Dry for 2–3 h in 30°C.
8. Transfer the colonies from the masterplates onto the YPD plates with the MOY457 cells using a replica plating device of choice.
9. Incubate the cells for 2 days in 30°C.
10. Replica plate the cells on YPG plates (requiring respiration) and incubate the cells for another 3 days. The respiration-competent colonies have obtained the pPT24-mtnLuc plasmid into the mitochondria and could restore *COX2* by homologous recombination between the plasmid and the mtDNA of MOY457. Positive colonies at this step are diploids.
11. For the next steps use the confirmed positive transformants from the SD-Leu plates, that are haploid (MOY160 positive haploid transformant). Troubleshooting 3.

**CRITICAL:** Carefully mark the masterplates and SD-Leu plates as well as replica plates to be able to find the corresponding positive transformants on them. These initial positive transformants from SD-Leu plates will be needed for the next steps (MOY160 positive haploid transformant).

### Induced recombination of mitochondrial DNA in a haploid strain via cytoduction

**Timing: 5 days**

These steps are required to obtain a haploid strain with the mitochondrial DNA containing the reporter construct. Here, the mitochondria are transferred into another strain by a process called cytoduction. This process depends on delayed karyogamy, which occurs in strains carrying the *kar1-*1 allele (Lancashire and Mattoon, 1979). During the cytoduction, exchange of cytoplasmic organelles occurs regularly, in our hands this process starts around 3 h 30 min after the two strains were mixed. On the other hand, nuclear genomes’ recombination is delayed by around 30 min. This timeframe between 3 h 30 min and 4 h allows for stopping the cytoduction, when the mitochondrial transfer occurred, but the original nuclear genomes are maintained. Homologous recombination of the mitochondrial *rho*^*+*^ genome of MOY457 strain and the plasmid of MOY160 pPT24-mtnLuc restores the *COX2* locus. The resulting strain is then a haploid that can be used to deliver the fully functional mitochondrial genome into a recipient strain of choice.

12. Start cultures of both the obtained MOY160 positive haploid transformant (carrying mitochondrial pPT24-mtnLuc) and MOY456 in 5 mL of YPD and grow for around 15 h at 30°C, 170 rpm.
13. The next day harvest (4,000 × *g*, 5 min) equivalent of OD_600_ 2 of both strains into a single tube.
14. Resuspend the cells in 100 μL of YPD and plate as a single drop on YPD plate.
15. Incubate the cells in an incubator at 30°C.
16. Transfer around a half of cells using a pipette tip or an inoculation loop to flasks with 10 mL YPD after around 3 h 45 min. Troubleshooting 4.

***Note:*** The incubation time may vary. For more information see problem 4 and possible solutions.

17. Incubate the cells in 30°C in a shaker (170 rpm) for 2–4 h.
18. Plate 50 μL of 20× diluted cell culture on YPG plates to get single colonies. Incubate in 30°C for 3 days.

***Note:*** The dilution factor in this step may vary depending on the strain’s growth speed and the number of cells taken in step 16. One can try different dilutions to obtain the right number of single colonies per plate.

### Screening for positive cytoductants

**Timing: 4 days**

19. Pick around 50 colonies from YPG plates with 3 h 45 min incubation time on YPD plates (masterplates) and incubate for 1 day at 30°C.

***Note:*** The colonies that could grow on respiratory medium plates (YPG) have restored the full mtDNA with mtnLuc, however they can be haploids or diploids.

20. The next day transfer the colonies using replica plating on SD plates lacking uracil (SD-Ura) and incubate the cells for 3 days in 30°C. This will allow to exclude haploid MOY456 cells.
21. The colonies that could grow on respiratory medium plates (YPG) and on SD-Ura can be the MOY160 mtDNA-mtnLuc haploid strains or diploid strains. To confirm the ploidy of the colonies one can use PCR approach to detect the mating types (Huxley et al., 1990) or mate colonies with a strain with mating type A and a different selection marker.

***Note:*** MOY160 (carrying mtDNA-mtnLuc and a nuclear *kar1-*1 mutation) can now be used in another round of cytoduction with a desired genomic background strain. This acceptor strain must be *rho*^*0*^ and mating type A.

***Note:*** If the final desired strain has a mating type α, it cannot undergo the cytoduction with MOY160 mtDNA-mtnLuc. In this case, other genetic manipulations are required, i.e mating of MOY160-pPT24-mtnLuc × MOY457 and tetrad dissection to obtain a spore with the correct mating type and *kar1-1* mutation. The spores obtained in this dissection can be used for another round of cytoduction to transfer the modified mitochondrial DNA to other strains of choice with different genetic backgrounds, i.e., W303 or BY4742.

## Expected outcomes

Reporter genes greatly contribute to studies on gene expression and protein function. Fluorescent or luminescent proteins are particularly popular and have been used in a variety of model organisms to shed light on processes as they occur in cells. For eukaryotic model organisms, expression of these reporters has been largely restricted to nuclear gene expression. Mitochondrial gene expression contributes a small set of subunits to the oxidative phosphorylation machinery. Hence, expression of genes encoded in mitochondrial DNA is important for cellular metabolism and organismal fitness, and needs to be coordinated with nuclear gene expression during metabolic reprogramming. One way to determine mitochondrial translation activity is to follow protein levels with Western blotting, however it may not be sensitive enough to detect small differences. Moreover, as the translation products need to assemble into large multimeric complexes and might be removed by proteolysis, when produced in excess or incorrectly, this readout via Western blotting is rather indirect. On the other hand, radiolabelling of newly synthesized mitochondrial polypeptides performed *in vivo* requires inhibition of cytosolic translation, which can affect cellular metabolism and mitochondrial translation. As a third alternative, profiling of mitochondrial translation can detect changes in translational efficiency, but the experiment is complicated and time consuming (Couvillion et al., 2016). The approach to integrate reporter genes into mtDNA is one solution to these problems and allows to study regulation of mitochondrial translation in a time-efficient, straight-forward way.

Here, we present a nanoluciferase system as the reporter for mitochondrial gene expression. What makes luciferases such attractive reporter enzymes is that there are no naturally occurring enzymes that emit light in most eukaryotes. Their activity can be measured and quantified using a variety of commercially available detection systems (e.g., Nano-Glo, Promega). In comparison to other luciferases, the genetically-engineered nanoluciferase (Hall et al., 2012) is both relatively small, more stable in denaturing conditions and upon pH changes (England et al., 2016). Moreover, it operates in an ATP-independent manner, which can be an advantage for studies on conditions resulting in changes of ATP levels in environment, like mitochondria.

**Figure 4:**
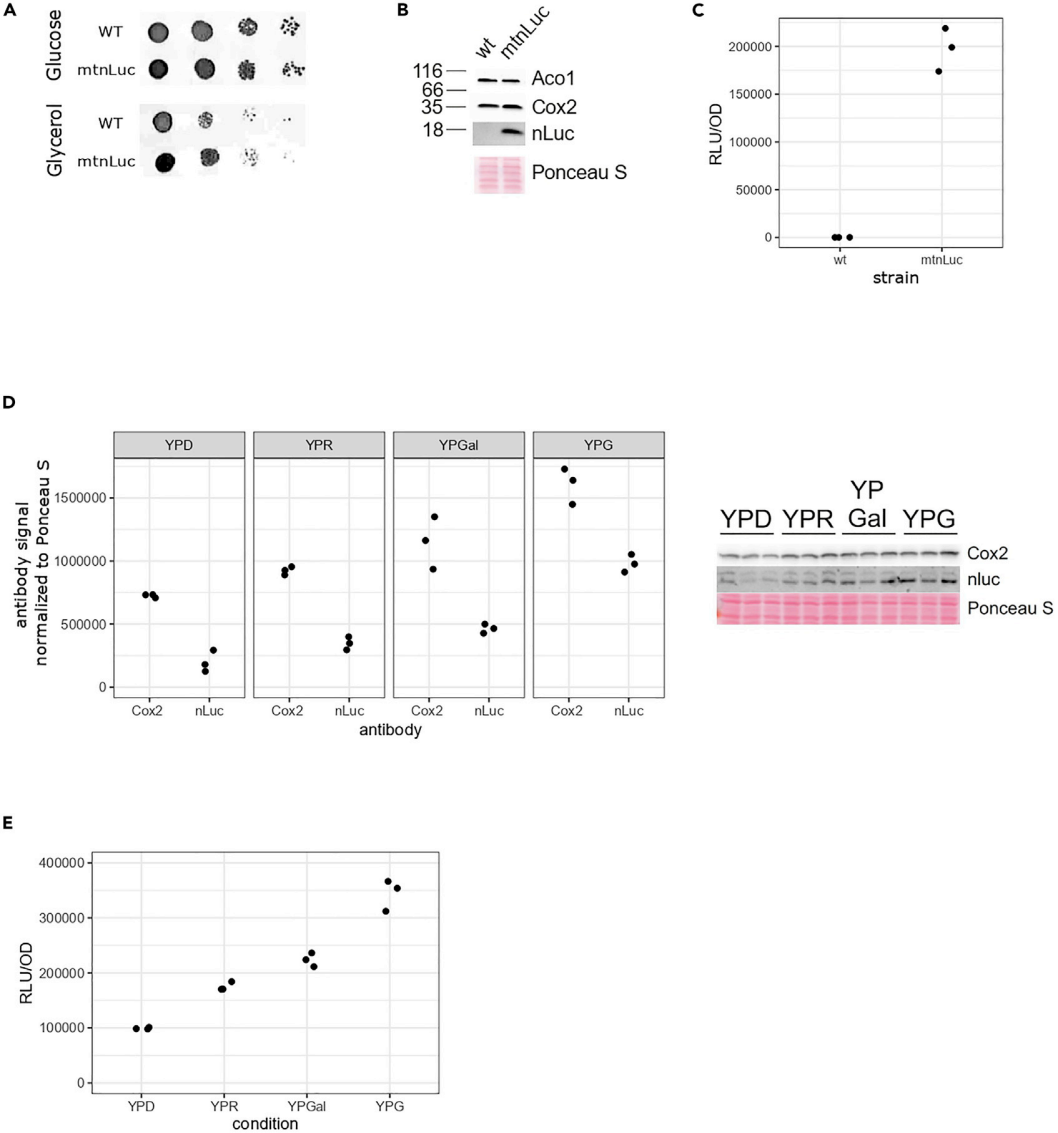
Functional characterization of the yeast strain expressing mtnLuc (A) Drop dilution test of yeast strains with wild type (WT) mtDNA and mtDNA expressing the reporter (mtnLuc) on non-respiratory (glucose) and respiratory (glycerol) media. (B) Proteins extracted from isolated mitochondria of WT and mtnLuc strains were separated on SDS-PAGE and analyzed with Western blot using indicated antibodies. (C) WT and mtnLuc strains were grown until exponential growth phase in YP media containing glycerol. Nanoluciferase signal was detected using a commercial kit (Nano-Glo, Promega) and normalized to optical density measured at 600 nm. (D) mtnLuc strain was grown in YP media with 2% of different carbon sources (YPD – glucose, YPR – raffinose, YPGal – galactose, YPG – glycerol) until exponential growth phase. Proteins were extracted and separated on SDS-PAGE and analyzed with Western blot using indicated antibodies (right). Quantification of the signal was based on three biological replicates and normalized to Ponceau S (left). (E) Nanoluciferase signal measured in the same samples as in (D) and normalized to OD_600_.

Following the protocol as detailed above, we integrated a recoded version of nanoluciferase (mtnLuc) into mtDNA. Expression of mtnLuc does not perturb mitochondrial function, as evidenced by robust growth on respiratory media containing glycerol (Figure 4A) and comparable levels of the mitochondrial proteins (Cox2 and Aco1) in mitochondria isolated from the WT and mtnLuc strains (Figure 4B). To confirm that the mtnLuc can be a useful reporter for mitochondrial gene expression, we followed the luciferase activity. With a readout at around 2 × 10^5^ of relative luminescent units (RLU) in the strain expressing mtnLuc and around 10^2^ of RLU in the wild type strain, we confirmed that the measured signal can be easily read and distinguished from the background (Figure 4C). Next, we compared the levels of mitochondrial gene expression following luciferase activity in yeast cells grown in different conditions. Yeast can utilize a variety of carbon sources to proliferate , each condition engaging distinct metabolic pathways. For example, cells incubated in media containing glucose do not require to perform oxidative phosphorylation and therefore biogenesis of subunits of the electron transport chain is repressed in a process termed glucose repression. In comparison, when cells are incubated in a carbon source like ethanol or glycerol that require respiration, increased mitochondrial gene expression is observed that leads to increased biogenesis of the OXPHOS complexes (Morgenstern et al., 2017).

To confirm that the mtnLuc can be used to detect changes in mitochondrial gene expression, we investigated mitochondrial protein levels in cells grown in media with different carbon sources and compared the results to mtnLuc activity (Figures 4D and 4E). Since the gene encoding mtnLuc is flanked with *COX2* UTRs and should follow similar expression pattern as *COX2*, we decided to follow Cox2 protein as the reference. We could see that both Cox2 and mtnLuc protein levels changed in a similar manner depending on the carbon source in the media. When measuring the luminescent signal in corresponding samples we could see the same changes in relation to the type of media. With that we confirmed that mtnLuc reflects mitochondrial protein synthesis levels.

In our hands, differences in luminescence levels can be much better quantified than results from the same experiment obtained with Western blotting. Likewise, accumulation of the normal mitochondrially encoded proteins requires their assembly into large, oligomeric complexes, which can be affected by multiple processes. Hence, a readout of a single, stable reporter will more faithfully reflect activity of mitochondrial gene expression. Another advantage of this reporter system is that the measurements can be obtained in significantly shorter amount of time, because less than five minutes are needed between taking the sample and the determination of the mtnLuc signal.

Due to the limited methods to study mitochondrial translation that often require e.g., protein extraction or cells’ fixing, immediate responses to different conditions can be difficult to follow. Mitochondrially encoded reporters like luciferases or fluorescent proteins (Suhm et al., 2018) can become advantageous tools for further studies on processes occurring in those organelles. The general approach described here offers further possibilities to interrogate mitochondrial protein synthesis. For example, mitochondrial encoded reporters for translational accuracy and read-through, inducible sequences leading to mitoribosomal stalling or other, more creative systems can be envisaged that have a great potential to unravel new aspects of mitochondrial biology.

## Limitations

One limitation is that the reporter system should not jeopardize mitochondrial functionality, as this will impact the different selection steps. This can be particularly important for proteins that challenge translation or protein folding inside mitochondria or that represent enzymes that interfere with substrates for the many reactions occurring inside mitochondria.

Biolistic transformation of the mtDNA has low success rate comparing to nuclear DNA transformation. In our hands only around 0.1% of the tested colonies contained the mitochondrial plasmid and could restore fully functional Cox2. It might therefore be advisable to start with at least 1,000 colonies for screening. We have also observed varying efficiency for cytoductions, which can require further testing to find the best time points, where enough haploid cells are obtained that carry recombined mitochondrial genomes.

## Troubleshooting

### Problem 1

Transformed cells after the biolistic transformation (step 4) may be too close to each other, which would make it impossible to pick up single colonies.

### Potential solution

Put the plate in the biolistic chamber on the lower shelf. That will allow the tungsten particles to spread more and penetrate cells on a larger area.

### Problem 2

Very few transformants are obtained after the biolistic bombardment.

### Potential solution

Several aspects can influence the transformation efficiency. It is essential to optimize the setting in the biolistic transformation device. Particularly important is the distance between the helium outlet in the chamber and the disruption disc. In our hands, a distance of roughly 0.5 cm works best. Moreover, the quality and correct quantification of plasmid preparations are essential.

### Problem 3

The success rate of biolistic transformation with the selection plasmid pRS315 is high, but low for penetrating mitochondria with pPT24-mtnLuc plasmid. If no colonies are positive after step 11, one has to test more colonies.

### Potential solution

Keep all the plates after the biolistic confirmation until the end of the whole procedure. In case of the lack of luck in the screening, one can test more colonies.

### Problem 4

The incubation time at step 16 may vary between different growth conditions and between the cells. The cells obtained after this step may be either unchanged or have undergone the nuclear DNA exchange (diploids), which would indicate the incubation was too short or lasted too long, respectively.

### Potential solution

We recommend trying different incubation time points. If no cells can grow on YPG, it means the mitochondria were not exchanged between the two strains and the incubation time should be extended. On the other hand, if all the colonies are diploids, the incubation time should be shortened. Depending on the problem, one can test different time points for transferring cells to liquid cultures. For example, start 30 min before or after the original time point 3 h 45 min and take samples every 10 min.

## Resource availability

### Lead contact

Further information and requests for resources and reagents should be directed to and will be fulfilled by the lead contact, Martin Ott (martin.ott@gu.se).

### Materials availability

Plasmids generated in this study have been deposited (Addgene ID numbers 184419 for pPT24 and 184339 for pPT24-mtnluc). Yeast strains can be shared upon request.

## Data Availability

This study did not generate any datasets or codes.
